# Abbreviated San Diego Wisdom Scale (SD-WISE-7) and Jeste-Thomas Wisdom Index (JTWI) – CORRIGENDUM

**DOI:** 10.1017/S1041610222000011

**Published:** 2022-03-23

**Authors:** Michael L. Thomas, Barton W. Palmer, Ellen E. Lee, Jinyuan Liu, Rebecca Daly, Xin M. Tu, Dilip V. Jeste

**Keywords:** Compassion, Spirituality, Loneliness, Well-being, Depression, Aging, Corrigendum

The above article (Thomas et al., 2021) was published with a few incorrect item numbers in the Results section and Table 1.

On the fourth page of the article, right-hand column, first paragraph, the last two sentences should read “Finally, for spirituality, the choice was between items 26 (“My spiritual belief gives me inner strength.”) and 25 (“There is no existence of the soul after death.”). We chose item 26 because it had a larger subscale discrimination parameter, had a shorter word length, and was judged to have better content validity (i.e., a greater focus on belief rather than on religious archetypes).”

In Table 1, first column, under “*Spirituality*,” the four entries should read “SDWISE 25”, “*SDWISE 26*”, “SDWISE 27”, and “SDWISE 28”.
